# Preventing type 2 diabetes: a qualitative study exploring the complexity of health-related practices in people with prediabetes

**DOI:** 10.3399/BJGP.2025.0208

**Published:** 2025-10-21

**Authors:** Eleanor Barry, Trisha Greenhalgh, Chrysanthi Papoutsi, Sara E Shaw, Anne Ferrey, Harry Rutter

**Affiliations:** 1 Nuffield Department of Primary Care Health Sciences, University of Oxford, Oxford, UK; 2 Department of Social and Policy Sciences, University of Bath, Bath, UK

**Keywords:** prevention, primary health care, public health, qualitative research, prediabetes, type 2 diabetes

## Abstract

**Background:**

Despite the introduction of primary care-based diabetes prevention strategies, labelling people with prediabetes and encouraging behaviour change, type 2 diabetes continues to rise, causing significant morbidity and mortality.

**Aim:**

To examine how a prediabetes diagnosis influences a person’s health-related practices.

**Design and setting:**

An in-depth qualitative study with 25 people with prediabetes, recruited via general practices.

**Method:**

The study included narrative interviews, patient-collected data, and follow-up interviews. Theoretical analysis was informed by Bourdieu’s theory of practice.

**Results:**

Participants with prediabetes, especially those from low-income and diverse ethnic groups, often had difficulty following prescribed ‘lifestyle’ recommendations. An individual’s habitus — that is, their embodied traits and behaviour patterns that had built up over their life—interacted with life–world influences, including the expectations and health beliefs of people in their immediate social circle (such as partner, children, and work colleagues); norms associated with wider social rituals (such as birthday parties); and structural intersectional influences (especially food availability and cost, influences of advertising, access to green spaces, and precarity, for example, housing insecurity). Going against social norms and expectations may risk an individual’s social positioning, cultural belonging, and sometimes job security. This risk was often experienced as more salient and pressing than a hypothetical future risk of diabetes.

**Conclusion:**

To improve the success of diabetes prevention efforts, interventions should go beyond individual-level behavioural advice to incorporate changes to the physical, economic, social, and cultural worlds that influence behavioural practices. By going against social norms ‘healthy’ behaviours may represent a personal social risk for some, particularly those from diverse ethnic groups.

## How this fits in

UK type 2 diabetes mellitus (T2DM) prevention efforts have focused on individual lifestyle change, but many patients, especially those from deprived or minoritised backgrounds, struggle to reduce their progression to T2DM. There is limited research on how social, cultural, and economic contexts influence patients’ ability to act on a prediabetes diagnosis. This study shows that behavioural practices are often constrained by patients’ life circumstances and social roles, alongside the fear of disrupting cultural norms. For GPs and public health practitioners, this highlights the need to move beyond standard advice and engage with the broader realities shaping patients’ health behaviours.

## Introduction

In the UK, 5 million people are living with diabetes, 90% of whom have type 2 diabetes mellitus (T2DM). A further 4 million people are estimated to be at high risk of T2DM.^1^ In the UK, 10% of the NHS budget is spent on diabetes and related conditions. The cost of managing T2DM is anticipated to rise to £39.8 billion by 2035/2036.^1,2^ T2DM is a complex social and medical condition whose risk factors include body mass index, socioeconomic status, deprivation, health literacy, ethnic group, age, and genetics.^3^ People with T2DM experience an estimated 6-year reduction in their life expectancy, with 52% of these premature deaths attributable to cardiovascular disease.^4^ Patients are at high risk of debilitating diabetes-related microvascular complications,^5^ this is particularly concerning given the rapid rise in T2DM incidence in teenagers and young adults from diverse cultural backgrounds. For this group, if their diabetes is not well controlled they will begin developing complications of T2DM 10 years after diagnosis (between the ages of 40 and 50 years).^6^

When a person’s blood test results show abnormal glycaemic biomarkers below the threshold for diabetes, that person will be given a diagnosis of ‘non-diabetic hyperglycaemia’, commonly known as ‘prediabetes’.^7^ GP practices in England are incentivised to identify and maintain a register of people with ‘non-diabetic hyperglycaemia’ (‘prediabetes’), undertake annual blood test monitoring, and offer referrals to behavioural interventions aimed at assisting the individual to reduce the progression to T2DM. Real-world evaluations of the NHS Diabetes Prevention Programme^8,9^ have shown that the intervention has helped many individuals improve their weight and glycosylated haemoglobin (HbA1c) if they are able to attend the intervention, engage in the full programme, and sustain behaviour change.^10^ However, evaluations have found low referral rates in areas of high deprivation, low engagement rates from diverse ethnic groups and women, and high attrition rates from referral to programme completion.^10–17^ This may be because of the ways in which individuals are informed of the prediabetes diagnosis and how this is internalised and contextualised.^18^

Little is known regarding how people from different backgrounds understand and internalise a prediabetes diagnosis, or how diabetes prevention messages are received in primary care or through prevention programmes.^19,20^ There is limited understanding of how people translate diabetes prevention messages into the context of their everyday health behaviours. Accordingly, we sought to explore the complexities that underpin health-related practices.^21^

The aim of this study was to explore how people interpret and internalise a prediabetes diagnosis, and to understand how people with prediabetes conduct health-related behavioural practices within the complexity of everyday lives. The research questions asked were:

- how do people react and respond to a prediabetes diagnosis?
- How does this diagnosis influence health-related practices? And
- how do sociocultural, economic, and environmental determinants influence behavioural practices relevant to type 2 diabetes prevention and how do participants navigate these?

## Method

### Sample and setting

The study included a sample of 25 people with prediabetes, ensuring a variety of ethnicities, socioeconomic statuses, wide age range, and a mix of female and male participants (Table 1). A specially designed text message was sent to approximately 1000 people with prediabetes registered at two participating general practices in North Central and North East London. A non-technical information sheet was emailed to interested participants outlining the purpose of the study and study requirements. The interviews (see Supplementary Box S1 for interview topic guide) were conducted online between October 2022 and February 2023; each lasting between 30 and 90 mins. With consent the interviews were recorded and transcribed verbatim. Participants did not receive payment for participating.

**Table 1.** Characteristics of study participants

Participant	Age, years	Gender	Cultural background	Country of birth	DM family history	Living arrangement	Accommodation	Occupation
1	42	F	Afro-Caribbean	Kenya	Father, grandmother, complications	Partner	Flat	Physiotherapist
2	40	F	Asian-Chinese	Indonesia	Father, mother, both grandparents. No insulin Siblings have pre-DM	Daughter	Flat	Doctor
3	57	M	Indian	India	Nil	Wife, two children, and daughter in law	House	Post office
4	60	M	Pakistani	Pakistan	Mother, GDM	Alone	House	Businessman
5	83	M	White British	England	Nil	Wife	Flat	Retired dentist
6	53	F	Chinese	UK	Mother, GDM	Partner and teenage stepdaughter	Flat	Civil servant
7	69	M	White British	UK	Father	Alone	Flat	Engineer
8	80	F	White British	England	Nil	Alone	Flat	Retired — finance
9	72	M	Declined to identify	London	Nil	Wife	House	Textile industry
10	52	F	Bangladeshi- American	New York	Mother, father, grandparents	Husband and two children	House	Full-time mum
11	72	F	White British	London	Nil	Alone	Flat	Ex-publican
12	78	F	White British	England	Nil (mother IHD)	Husband	House	Retired teacher
13	80	M	White British	England	Nil	Wife	House	Carer (retired engineer)
14	63	F	Iranian	Iran	Father DM, mother IHD	Husband and daughter	House	Carer and volunteer
15	76	M	White British	England	Nil	Alone	Flat	Retired architect
16	74	F	White British	England	Nil	Partner	House	Media/retired
17	68	M	White British	England	Father – HTN CVD <60 years	Wife and son	House	Finance
18	83	F	Jewish	South Africa	Nil	Alone (widow)	Flat	Retired accountant
19	64	M	White British	England	Mother and father	Alone	Flat	Retired accountant
20	78	F	White British	Scotland	Nl	Alone	Flat	Retired publisher
21	89	F	Jewish	Germany	Nil	Alone	Flat	Retired insurance
22	52	F	Iraqi-American	Florida	Nil	Alone (partner in France, son lives in London)	Flat	Musician
23	77	F	White	Scotland	Nil	Husband	House	Retired
24	63	M	White	England	Nil	Sister	House	Engineer
25	69	M	Jewish	London	Nil	Wife	Flat	Retired doctor

CVD = cardiovascular disease. DM = diabetes mellitus. F = female. GDM = gestational diabetes mellitus. HTN = hypertension. IHD = ischaemic heart disease. M = male.

### Research techniques

This qualitative study of people with a diagnosis of ‘prediabetes’ had three components:

- initial narrative interview;
- cultural-probe exercise; and
- reflective interview.

#### Initial narrative interview

A narrative interview allows the individual to tell their story in an unstructured way.^22^ Here we explored how the person responded to and interpreted a ‘prediabetes’ diagnosis, and how this response influenced behavioural change. These interviews provided insights into how NHS prevention policies and wider public health policies influenced individual health practices.^23–25^

#### Cultural-probe exercise

Cultural probes are artefacts collected by participants that reflect what is salient to them in the context of their everyday lives, allowing participants to be their own researchers.^21,26,27^ Participants were instructed to collect information on what and who was important to them, as well as information on their favourite meals and pastimes. Data collected included food diaries, screen shots of exercise apps, pictures of food, cultural activities, family and friends, shopping receipts, and menus from eating out (see Supplementary Figures S1–S6 for examples). This exercise provided further contextual data and insights into the competing priorities the patients experienced.

#### Reflective interview

In this final interview, the participants reflected on and explored the data they had collected. Discussions structured around the cultural probes enabled the participant and interviewer to co-construct a narrative about how behavioural practices were shaped, facilitated, and constrained by the person’s immediate and wider social, cultural, and material context.^25^

### Data analysis and theoretical approach

An interpretivist approach was taken to examine the social realities that exist for people from different backgrounds. An abductive analysis was conducted, applying theoretical perspectives to the datasets in an iterative process, refining the synthesis as the analysis progressed.^28–31^ The interviews were conducted by the first author, a GP and DPhil candidate, whose research focused on diabetes prevention strategies. The first author adopted a reflexive approach, actively challenging preconceptions under the guidance of the supervisory team who have clinical, public health, and social and behavioural science backgrounds (the other authors).

The first (descriptive) stage of analysis involved close reading and examination of each narrative to understand what was salient for that participant as part of their story. NVivo was used to manage the large volume of data. Each transcript was initially coded thematically. Coded excerpts from across different interviews were compared, combined, and grouped into themes.^21^ Themes were refined iteratively using constant comparison of the data from each source.^21,31,32^

Bourdieu’s theory of practice was used, particularly the concepts of habitus and capital to theorise how participants responded to a diagnosis of prediabetes, with a focus on how the diagnosis became embedded within an individual’s habitus and how health practices were socially constructed.^33^ Habitus refers to the dispositions to act that are shaped over time by cultural backgrounds, social contexts, role and expectations, upbringing, and education.^34^ Health-related practices such as eating, exercising, and engaging in physical activity are not simply physiological acts but socially embedded behaviours shaped by the interaction of an individual’s habitus with intersecting social and community contexts.^33,35,36^ Three interrelated social contexts influencing habitus were identified:

- the immediate sociocultural environment of the home and family;
- wider societal and cultural norms; and
- community structural influences.

**Figure 1.**
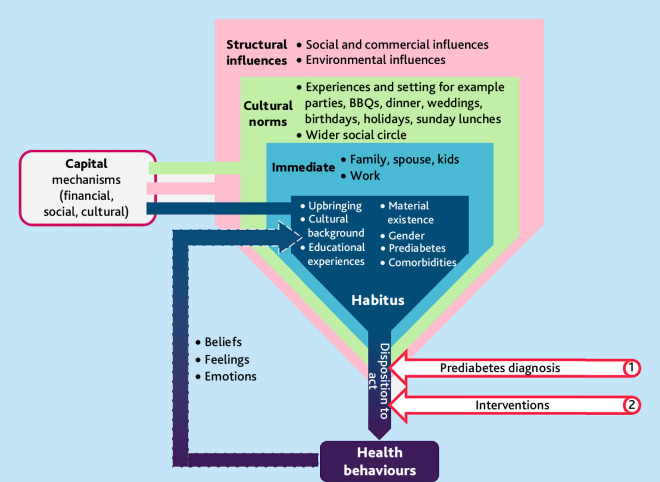
An adapted Bourdieu’s theory of practice for people with prediabetes

These contexts not only shaped and constrained behaviours but also interacted dynamically with them. Health-related practices emerged from, and over time contributed to, reshaping these environments (see Figure 1). Bourdieu’s notion of capital (economic, social, and cultural) further illuminated how individuals and communities made sense of and responded to expectations around behaviour change, influencing their capacity to mitigate the risk of developing T2DM within a wider political and economic landscape.

## Results

### Description of sample

In total, 14 females and 11 males took part in the study, ranging in age from 40 to 89 years and spanning a wide variety of cultural backgrounds and professions (Table 1). Twenty-five participants took part in the initial interview; 21 participants completed the follow-up interview and 17 participants collected cultural-probe data. For participants who did not collect cultural-probe data the second interview focused on their feelings and reflections about navigating their risk state.

### How participants experienced the prediabetes diagnosis

Participants understood prediabetes as a medical condition defined by a numerical value outside a normal range, typically linked to behavioural factors. They particularly valued in-person consultations with clinicians who took time to understand their social and familial contexts. In the absence of such interactions, participants often felt abandoned by the system, as illustrated by one participant who described the anonymity of telephone consultations and the lack of continuity of care:

*‘I did adjust my diet, because everything was done over the phone I felt like slightly abandoned by the system … it just seems very anonymous. I never really knew my GPs because the system is like you deal with different GPs.’* (Participant [P]10, Bangladeshi-American, female [F])

Most participants saw it as their responsibility to change their behaviours to reduce the risk of developing T2DM, but many struggled to make lasting changes owing to uncertainty about how much they needed to alter. This reflects a liminal state, participants were no longer fully well, but not yet unwell, navigating an ambiguous ‘at-risk’ position, often marked by fear of progression to T2DM and little clear guidance. Participant 17 described the diagnosis as:

*‘They tell you it’s above the completely normal range but it’s not ... at a level that requires any intervention ... it’s sort of an amber light where you’ve got to be a bit more careful.’* (P17, White British, male [M])

In contrast, when imagining a T2DM diagnosis, this same participant said:

*‘I would immediately lose weight, cut out sugar and carbohydrates, and start taking more exercise.’* (P17, White British, M)

This distinction between cautious monitoring with prediabetes and decisive action in response to T2DM was common across interviews. The uncertainty surrounding the label often led to hesitation about how to respond. These responses were shaped by participants’ habitus, socially and culturally embedded dispositions that influenced how they interpreted risk and responsibility. Some engaged in self-surveillance (such as tracking activity, see Supplementary Figure S1) to manage the uncertainty of T2DM development. These practices reflected internalised expectations and dispositions to act grounded in their habitus.

### Perceived social influences on behaviour practices

#### The home and family

Family dynamics strongly influenced participants' eating practices, reflecting the embodied dispositions of habitus, shaped over time by gender roles and caregiving expectations. Dietary changes were easier when partners and children were supportive, but resistance from family members often led to conflict, making adherence more difficult. For example, one female described repeated opposition from her daughter and husband when trying to reduce calorific foods at home:

*‘Our daughter loves a lot of food with sauces and things which I cannot eat and … it becomes quite an issue, that I’m making a lot of trouble at home.’* (P14, Iranian, F)

Consequentially participants compromised their health priorities to avoid domestic conflict, with some preparing separate meals or ordering takeaways for other family members. Many prioritised their family’s dietary needs over their own, particularly when caring for children or relatives who were old.

Gendered expectations of caregiving were a recurring theme in the interviews, highlighting how habitus is shaped by social roles that disproportionately assign care work to women. This unequal burden limited participants’ ability to focus on their own health behaviours, such as diet and exercise. As one participant described:

*‘I think between COVID, having the kids home for 2 years practically non-stop and then, I was literally like a waiter ... I think I’m totally out of sync now and it’s kind of torture to break out of it mentally.’* (P20, White British, F)

Here, being ‘out of sync’ captures her struggle to re-establish routines around self-care, illustrating how deeply internalised gendered expectations can hinder efforts to maintain health-promoting practices.

#### Historical cultural influences

Participants often referred to childhood food practices and cultural traditions that continued to shape their current eating practices. These practices reflect the lasting influence of cultural capital formed through early familial socialisation. Many recalled str0ng cultural and family links, such as cooking or growing vegetables with grandparents, which carried nostalgic associations and symbolised familial love. These rituals also fostered a sense of identity and belonging, reinforcing the continuity of cultural traditions into the present (see Supplementary Figure S3) and as outlined by this participant:

*‘My grandmother was very loving and was a great cook ... I kind of picked that up and associated that with her. And my father … was desperately trying to grow his own veg because he loved feeding his own family with fresh vegetables, I realise the extent to which my own attitudes to food still reflect that.’* (P6, Chinese, F)

### Wider sociocultural influences

Social events, such as restaurant outings and religious, familial, and cultural celebrations, influenced participant’s food-related behaviour (see examples in Supplementary Figures S4–S6). These rituals carried symbolic significance, reinforcing community belonging and cultural capital. Many found it difficult to refuse high-calorie foods during these events owing to implicit social and cultural expectations in each social setting. For example, one participant chose chips and a steak sandwich to match his son and grandsons’ orders at a pub, illustrating the influence of context-specific social norms. For many the risk of social exclusion often outweighed the concerns of consuming unhealthy foods or alcohol.

In the workplace, food similarly facilitated social bonding and relationship building with pressure to maintain symbolic belonging to secure social capital. One participant referred to navigating a ‘sugar gauntlet’ to reach the kettle, highlighting the challenge of sustaining dietary change (see, for example, Supplementary Figure S2). Office workers also described a ‘cake culture’, and a near constant supply of treats as part of sharing celebrations with colleagues. Some feared that opting out of such practices or disclosing health conditions could affect workplace relationships or career prospects, reinforcing pressure to conform:

*‘When it comes down to who we are gonna choose to promote ... if they’re equal candidates, are we gonna choose the person who’s ill? ... You can understand why ... staff don’t talk about their conditions a lot.'* (P19, White British, M)

Participants navigated competing pressures: while they understood the biomedical rationale for changing their diet to prevent diabetes, powerful cultural, familial, and social influences often hindered this shift. Bourdieu’s concept of cultural capital is central to understanding this tension. Conforming to food-related norms, such as eating traditional dishes at family or workplace events, reinforced participant’s social identity and maintained their status within their social group. In these contexts, maintaining cultural capital and a sense of belonging often took precedence over potential long-term health considerations.

### The community environment: material, relational, and financial influences

#### Community relationships

Personal relationships with local shop keepers and market vendors were socially protective for many participants, especially those from diverse ethnic groups, who valued access to culturally appropriate foods. These relationships fostered a sense of community belonging:

*‘Because we, we wanted them still to be there and very much, you know, the owner works in there, they’re super friendly, they remember who you are so I don’t know if I kind of captured those in those photographs but they will remember who you are and what your order is and if they’re not completely run off their feet, you’ll have a chat while they’re making it and it’s a proper kind of local business so I actually felt my enormous breakfast of spaghetti bolognese were contributing to the community because they were supporting a small business.’* (P6, Chinese, F)

#### Commercial food environment

Participants emphasised the need for accessible, affordable, and healthy food outlets in their communities. Those living in low-income areas noted the scarcity of these options, with fast food chains more prevalent than health-focused alternatives:

*‘I could go to Pret* [a Manger] *if I wanted to … I’d more likely eat at home or make something or go to Tesco’s and get supplies that kind of thing. I think it’s that kind of balance because if I wanted to there’s like four fried chicken places, I could go to.’* (P1, Afro-Caribbean, F)

Participants with children described using app-based food delivery services to accommodate differing preferences, reflecting how food technology has shifted family meal-time norms away from shared preparation and communal eating practices.

#### Cost of living and affordability of health food

Rising food and household costs made it difficult for participants with limited financial means to prioritise healthy eating. Here, Bourdieu’s notion of economic capital becomes particularly salient, shaping, enabling, or constraining dietary choices. One participant described having to forgo fresh ingredients in favour of cheaper, processed meals:

*‘I have been known to buy because I worked it out the other day there was something in Tesco, what was it? It was cauliflower cheese and it was something like £1.65 and I thought, I couldn’t make it for that.’* (P8, White British, F)

This disparity was particularly evident when comparing participants with differing economic resources: those with higher incomes reported easier access to nutritious food and opportunities for physical activity, whereas those with fewer financial resources faced greater barriers to adopting healthy practices:

*‘We have a fishmonger we’ll get fish from there or from the farmer’s market which might be tuna steaks or cod and then we’ll have that and we usually get me some smoked salmon for some of my lunch if I’m, if I’m feeling it that week. Yeah, and so farmer’s market, lots and lots and lots of veg and fruit and stuff but the main shop is the big Sainsbury’s and stuff that yeah, I would say it’s pretty expensive to live the life I live.’* (P1, Afro-Caribbean, F)

#### Health literacy

One participant's quote illustrates how health literacy can support informed decision making within social contexts:

*‘Yesterday when we went to the pub for lunch, it’s Sunday, I want a Sunday roast, usually I have the pork belly, that’s my favourite roast, 2600 kcal was on the menu. It just turns you off right away, I ended up having the burger and chips which was like 1600 kcal or something. But I think the fact it’s there in your face, makes you rethink your options. Even pudding, sticky toffee pudding was 900 kcal, I know! 900 kcal for a sticky toffee pudding with ice cream. It’s like a day and a half’s calories if you had chosen that. So I had the brownie instead which was like 650 kcal.’* (P2, Asian-Chinese, F)

Drawing on Bourdieu’s theory of practice, their ability to interpret and act on calorie information reflects the dispositions shaped over time by social and educational experiences, deeply embedded in an individual’s habitus. Rather than withdrawing from the shared social practice of eating out, the participant adapted their choices to align with their health goals and the social context. This highlights the critical role of health literacy, shaped by structural and social conditions, in supporting diabetes prevention.

#### Green spaces and safe housing

Access to outdoor spaces and quality housing further illustrates how economic capital materially shapes the ability to engage in health-promoting behaviours:

*‘Yeah we’ve got some fruit trees in the garden and my, yeah I live in a flat that my friend, my neighbour below me she’s a close friend and we collect the fruit, we do a lot of the garden, I do a lot of gardening.’* (P15, White British, M)

*‘I'm having a lot of stress with my landlord, there are unsafe cracks and leaks around the main building.’* (P15, White British, M)

Participants with greater economic resources, typically from more affluent backgrounds, described the mental and physical health benefits of living near green spaces, which supported regular exercise. They also had more flexibility to make healthier choices, including purchasing fresh produce, joining fitness classes, and accessing respite care. In contrast, those from lower-income backgrounds often faced structural constraints, such as poor housing and limited access to safe, well-maintained public spaces. These conditions had a negative impact on mental health and reduced an individuals’ capacity to prioritise their wellbeing.

Consistent with Bourdieu’s theory, economic capital operated at both individual and community levels, shaping not only individual-level practices but also the wider neighbourhood context, including safety, housing quality, food access, and proximity to green spaces, all of which influence capacity for behaviour change. As illustrated in Figure 1, Bourdieu’s framework helps us to explain how behaviour choices are embedded in social practices and material conditions. Participants’ experiences reflected the dynamic interplay of agency with cultural, social, familial, and economic influences.

#### Discourse on diabetes prevention

Despite barriers arising from structural constraints, those unable to reduce their weight or HbA1c often internalised blame, attributing it to a lack of willpower or discipline:

*‘I mean who else, who else is responsible … I think it’s just the discipline, I lack discipline.’* (P9, declined to identify cultural background, M)

*‘You know, I can wake up with the best intentions in the world and then find all sorts of reasons why I’m not gonna do it* [exercise]*. So, I’m afraid it’s just weakness of character and self-discipline. I’m not going to cure that anytime soon.’* (P10, Bangladeshi-American, F)

This misrecognition obscures the unequal distribution of capital and reinforces dominant societal narratives that frames T2DM as an issue of individual responsibility, despite the substantial influence of structural factors. T2DM must be contextualised within broader social, cultural, commercial, political, and economic systems that shape health behaviours, rather than a consequence of individual biological failure.

## Discussion

### Summary

In this study we have explored how participants with prediabetes understand the diagnosis and undertake health-related practices in the context of their daily lives. Primary care diabetes prevention policies assume linear behaviour change, but in reality, this is far more complex. Participants with prediabetes felt it was their own responsibility to reduce their risk of developing the condition, even when they felt limited in their ability to do so. Their behaviour choices were influenced by their habitus, social contexts, and capital (financial and cultural), and wider social and commercial determinants of health.

Those most able to change their behavioural practices and overcome structural barriers were participants who were supported by primary care teams and whose habitus and social context already aligned with these interventions. Participants were better able to adapt their behavioural practices if they had a supportive spouse and family to help maintain these changes and sustain them across settings. People also required the financial capital to afford a healthy diet while living in an area in which healthy food options were convenient and accessible. Many struggled to navigate social contexts; avoiding social disruption felt more pressing than a hypothetical future risk of developing T2DM.

**Figure 2.**
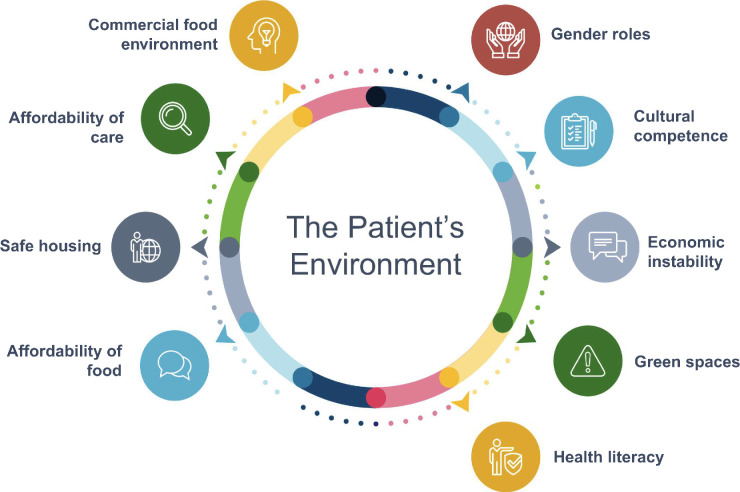
Intersectional influences on health-related behaviours

Our analysis suggests that the social and commercial determinants of health are intersectional in nature. Intersectionality is a framework that explores how cumulative individual repetitive experiences (of structural influences) become ‘interlocking’, which in this analysis may explain why behaviour change was insurmountable for some people.^37,38^ Some of the intersectional influences are outlined in Figure 2. For example, in our study, women from diverse ethnic groups described societal sexism, being conditioned to place their needs last as caregivers, and also experienced cultural unawareness exhibited by the incorrect assumptions of their social worlds by health practitioners. The greater the number of intersecting structural barriers, the less likely the participant was able to engage in long-term behaviour change. This may explain why women have been found in evaluations of interventions to be less able to reduce their risk of diabetes.^11^ Participants who had supportive social capital (in the form of a supportive spouse and family), good health literacy, or financial capital (with the ability to afford healthy food options) could negate some of these influences.

The prevention of T2DM is a multifaceted challenge that is far more complex than a simple diagnosis or prescribed lifestyle change. The wider environment and commercial determinants of health play a critical role in shaping the practices that contribute to diabetes development. For instance, the food industry has an impact on consumption patterns through the pricing of unhealthy food options, the strategic placement of outlets in communities (or online), and the cultural normalisation of these. Moreover, technological advances extend the convenience of unhealthy foods directly into homes. These tools allow families to order multiple meals from different delivery services through the same app, illustrating how technology can exacerbate unhealthy consumption habits. In an environment where unhealthy commodities are widely available and actively promoted, the wider community health system inadvertently supports the dominance of these industries. Easy access to cheap, ultraprocessed, addictive food makes it unjust to place the burden of the prevention of T2DM solely on individuals.^39–41^

### Strengths and limitations

This study provides an in-depth qualitative exploration of how people understand and respond to a prediabetes diagnosis. By engaging with participants over repeated meetings and the collection of cultural-probe data, increased depth and longitudinal insights into their lived experiences and their reflective practices was given. The inclusion of a diverse sample enhanced the breadth of perspectives gathered. The application of a theoretical lens gave a robust framework, enabling us to make sense of participants’ experiences in a structured and conceptually grounded way.

However, the study sample was less diverse than intended, owing in part to recruitment challenges following the COVID-19 pandemic, which may limit the applicability of findings to underrepresented groups. Although the study acknowledges the role of commercial determinants of health, there was insufficient opportunity to expand on this.

### Comparison with existing literature

Health-related behaviours are the result of a dynamic interplay between all the different components of Bourdieu’s theory of practice, helping to explain why at different times and in different contexts, people make different health-related decisions. Similar to findings from Hindhede’s ethnography of people with prediabetes, this in-depth study suggests that people navigate their risk of T2DM while also considering the impact on their social lives.^20^ Hindhede’s qualitative study also found that people’s health-related behaviours were strongly influenced by other people at home and at work; however, this study was situated in a randomised control trial with a trial population actively engaging in behaviour change. This study included people who had been in lifestyle interventions previously but focused more on how the learning had been incorporated into real-world settings. Howells and colleagues' qualitative study of people with prediabetes showed that people strongly resisted the diagnosis and associated behaviour changes because of their social contexts.^18^ Similarly, the participants in this study resisted behavioural change due to the pressures of social norms. In contrast, this study found that although people accepted the biomedical diagnosis, they struggled to respond because of the liminality of the precondition, as well as social, economic, and environmental barriers.

Another qualitative study by Twohig *et al* identified several barriers that people faced when trying to reduce their T2DM progression, which aligns with the findings of this study.^19^ This study builds on the findings of Twohig *et al* by applying Bourdieu’s theory of practice to deepen understanding of how individuals respond to being informed of their risk condition and how they navigate health-related behaviours within diverse social, economic, and environmental contexts.

### Implications for research and practice

This study highlights the need to reframe T2DM prevention from a focus on individual responsibility to one of shared societal responsibility.^42–44^ Central to this is recognising that T2DM is not simply a biological failing, but a complex condition resulting from the interaction of individual characteristics and structural, environmental influences.

In primary care, a holistic, relationship-based approach is essential when supporting patients at risk of T2DM. The prediagnosis phase is often marked by uncertainty, fear, and stigma. Primary care practitioners are well positioned to explore the broader social, economic, and cultural contexts that influence a person’s capacity for behaviour change. Recognising these intersecting influences such as financial insecurity, time constraints, or limited access to healthy food and safe exercise environments can support tailored and realistic risk reduction strategies with patients' lived realities.

At a health-systems level, the success of diabetes prevention also relies on a coordinated and adequately funded public health infrastructure. Integrated care systems aim to foster place-based partnerships between integrated care boards (ICBs) and local authorities to tackle population health challenges. However, with the abolition of NHS England and the reorganisation of services under neighbourhood health models, primary care teams will operate within new local structures, while public health responsibilities remain within ICBs. Simultaneously, ICBs have been instructed to reduce their running costs by 50%.^45^ This combination of organisational fragmentation and significant funding reductions risks undermining an interdisciplinary, collaborative approach to the prevention of T2DM, obesity, and other long-term conditions.^46–52^

Individual-level diabetes prevention strategies must be partnered with robust public health action to address the broader social and commercial determinants of health. Public health teams require adequate funding and structural support to deliver upstream population-level interventions. National government also has a critical role in facilitating system change ensuring access to affordable, healthy food, improving health literacy, enhancing housing and working conditions, investing in safe green spaces, supporting affordable care options, and regulating the commercial food environment. Without such systemic action, T2DM will continue to disproportionately affect the most disadvantaged, entrenching existing health inequities.

Future research should apply systems thinking, grounded in complexity science, to identify effective intervention points at both community and population levels. This approach can complement individual-level strategies by addressing the interdependent and upstream determinants of health behaviours. Further research is needed to examine how commercial determinants, such as food industry marketing, digital technologies (including artificial intelligence), urban planning, and economic policies interact to shape T2DM risk and inform more equitable prevention strategies. A systems approach also requires coordinated, cross-sectoral action involving public health, local government, education, industry, and community organisations. Future studies should explore how these sectors can work collaboratively to codesign sustainable, contextually appropriate, and equitable interventions, especially for communities most affected by T2DM.

In conclusion, this study underscores that health-related practices are socially constructed and shaped over the life course through complex interactions within individuals' home, social, community, and broader environmental contexts, including the influence of commercial actors such as multinational food corporations. A prediabetes diagnosis becomes embedded in an individual’s habitus, shaping behavioural dispositions in relation to these contexts. Our findings challenge the dominant biomedical model that places the burden of behaviour change solely on individuals, minimising the structural and social complexities involved. This study highlights the need for a holistic, systems-based approach to diabetes prevention, one that acknowledges the influence of social networks, housing, local resources, and wider environmental and commercial factors. For GPs, public health teams, and policymakers this means designing interventions situating the responsibility of disease prevention within the broader community and national contexts, supporting the development of environments that promote sustainable and equitable health-related practices.
